# Extended genomic HLA typing identifies previously unrecognized mismatches in living kidney transplantation

**DOI:** 10.3389/fimmu.2023.1094862

**Published:** 2023-01-27

**Authors:** Claudia Lehmann, Sarah Pehnke, Antje Weimann, Anette Bachmann, Katalin Dittrich, Friederike Petzold, Daniel Fürst, Jonathan de Fallois, Ramona Landgraf, Reinhard Henschler, Tom H. Lindner, Jan Halbritter, Ilias Doxiadis, Bernt Popp, Johannes Münch

**Affiliations:** ^1^ Institute for Transfusion Medicine, University Hospital Leipzig, Leipzig, Germany; ^2^ Division of Nephrology, Department of Internal Medicine, University of Leipzig Medical Center, Leipzig, Germany; ^3^ Division of Visceral Surgery and Transplantation Medicine, University of Leipzig Medical Center, Leipzig, Germany; ^4^ Department of Pediatric Nephrology, University of Leipzig Medical Center, Leipzig, Germany; ^5^ Institute of Transfusion Medicine, University of Ulm, Ulm, Germany; ^6^ Department of Nephrology and Medical Intensive Care, Charité Universitätsmedizin Berlin, Berlin, Germany; ^7^ Institute of Human Genetics, University of Leipzig, Leipzig, Germany

**Keywords:** kidney transplantation, HLA typing, HLA mismatch, donor specific antibodies, NGS, epitope matching

## Abstract

**Introduction:**

Antibody mediated rejection (ABMR) is the most common cause of long-term allograft loss in kidney transplantation (KT). Therefore, a low human leukocyte antigen (HLA) mismatch (MM) load is favorable for KT outcomes. Hitherto, serological or low-resolution molecular HLA typing have been adapted in parallel. Here, we aimed to identify previously missed HLA mismatches and corresponding antibodies by high resolution HLA genotyping in a living-donor KT cohort.

**Methods:**

103 donor/recipient pairs transplanted at the University of Leipzig Medical Center between 1998 and 2018 were re-typed using next generation sequencing (NGS) of the HLA loci -A, -B, -C, -DRB1, -DRB345, -DQA1, -DQB1, -DPA1, and -DPB1. Based on these data, we compiled HLA MM counts for each pair and comparatively evaluated genomic HLA-typing with pre-transplant obtained serological/low-resolution HLA (=one-field) typing results. NGS HLA typing (=two-field) data was further used for reclassification of *de novo* HLA antibodies as “donor-specific”.

**Results:**

By two-field HLA re-typing, we were able to identify additional MM in 64.1% (n=66) of cases for HLA loci -A, -B, -C, -DRB1 and -DQB1 that were not observed by one-field HLA typing. In patients with biopsy proven ABMR, two-field calculated MM count was significantly higher than by one-field HLA typing. For additional typed HLA loci -DRB345, -DQA1, -DPA1, and -DPB1 we observed 2, 26, 3, and 23 MM, respectively. In total, 37.3% (69/185) of *de novo* donor specific antibodies (DSA) formation was directed against these loci (DRB345 ➔ n=33, DQA1 ➔ n=33, DPA1 ➔ n=1, DPB1 ➔ n=10).

**Conclusion:**

Our results indicate that two-field HLA typing is feasible and provides significantly more sensitive HLA MM recognition in living-donor KT. Furthermore, accurate HLA typing plays an important role in graft management as it can improve discrimination between donor and non-donor HLA directed cellular and humoral alloreactivity in the long range. The inclusion of additional HLA loci against which antibodies can be readily detected, HLA-DRB345, -DQA1, -DQB1, -DPA1, and -DPB1, will allow a more precise virtual crossmatch and better prediction of potential DSA. Furthermore, in living KT, two-field HLA typing could contribute to the selection of the immunologically most suitable donors.

## Introduction

In patients with chronc kidney failure, kidney transplantation (KT) is the therapy of choice as it increases quality of life and reduces morbidity and mortality compared to patients who remain on hemodialysis (1). Though the transplant procedure itself is expensive, the economic burden is lower than in patients who continue to receive kidney replacement therapy (2, 3). As the waiting time for a deceased donor graft varies, depending on the countries transplant legislation and the Organ Procurement Organization (OPO), living-donor KT allows for reduced time on dialysis and optimized patient’s outcome. If kidney transplantation can be accomplished, subsequent preservation of organ function is of paramount importance. In general, chronic antibody mediated rejection (ABMR) is the most important cause of premature graft loss in both deceased and living donor KT. Despite the use of immunosuppressive agents, the probability of developing donor-specific HLA antibodies (DSA) is associated with the number of mismatches (MM) in human leukocyte antigens (HLA) patterns, and subsequently with an increased risk of both graft rejection and graft loss (4–7). The management of ABMR is challenging, as there are no therapeutic options that have been proven to substantially improve transplant outcomes.

Therefore, adequate definition of HLA-A, -B, -C, -DRB1, -DRB345, -DQA1, -DQB1, -DPA1, and -DPB1 geno- and pheno-types is essential for organ allocation. Allocation organizations or OPO still refer in their registry to HLA typing on the antigen level and additional use of (un)acceptable HLA antigens due to pre-existing HLA antibodies in the recipient (8). However, recent studies delineate that the recognition of only few dissimilar amino acids by the recipient’s immune system are sufficient to provoke humoral allosensitization (9). The application of so-called “broad” HLA antigens for the determination of suitable donor/recipient pairs, established to include the contribution of sensitization, seems outdated and inaccurate. Next generation sequencing (NGS) techniques represent a cost-efficient and rapid way to provide two or more field resolution HLA typing, allowing MM to be detected at the more precise amino acid level.

**Figure 1:**
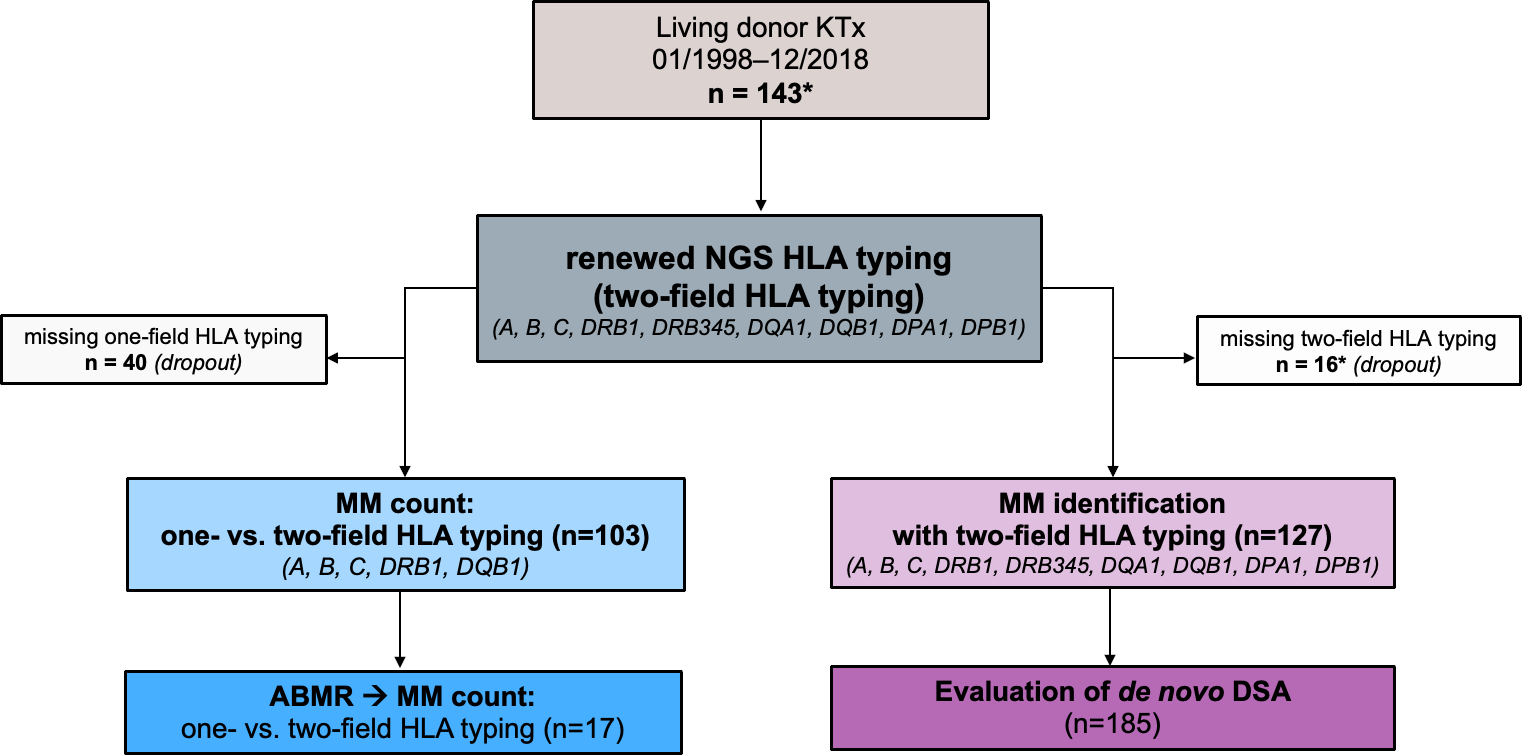
Study design. A total of 143 donor/recipient pairs who underwent living kidney transplant at the University of Leipzig Medical Center between 1998 and 2018 were enrolled for the study (*of note, one patient received two consecutive living kidney transplants - donors were the father and mother, respectively). The HLA typing data of 103 of these donor/recipient pairs were available in the registry at one-field resolution (i.e., serological HLA typing or low-resolution genotyping). Based on these data, a mismatch count for HLA A, B, C, DRB1, DQB1 was generated and compared with one obtained from two-field HLA typing (NGS HLA typing). For 127 of the donor/recipient pairs, we had in-center data from two-field HLA typing for 11 HLA loci A, B, C, DRB1, DRB3, DRB4, DRB5, DQB1, DPA1, DPB1). Based on the results, we showed the frequency of allele-level HLA mismatches in our cohort and analyzed how often HLA antibodies can be classified as donor-specific. ABMR, antibody mediated rejection; DSA, donor specific antibodies; HLA, human leukocyte antigen; KTx, kidney transplantation; MM, mismatch(es).

To assess the impact of precise NGS HLA typing methods, we compared the MM load generated by one-field resolution and/or serological typing done previous to transplantation with repeated NGS HLA typing in a single center cohort. Based on these results, we investigated the frequency of missed HLA MM. In addition, we determined the frequency of HLA MM in HLA loci previously unconsidered for allocation in some OPO’s (i.e., DRB345, DQA1, DPA1, DPB1) including their effect on *de novo* DSA formation (**Figure 1**).

## Methods

### Probands

This study includes donor/recipient pairs who underwent living donor KT at the University of Leipzig Medical Center (Germany) between January 1998 and December 2018. Patients were transplanted only in case of a negative complement dependent cytotoxicity (CDC) crossmatch between donor and recipient prior to transplantation. Written informed consent was provided by all patients and donors participating in this study. The study was approved by the local institutional review board (IRB) at the University of Leipzig, Germany (ethics vote 504/21-ek).

### DNA-isolation for genetic HLA typing

Genomic DNA was isolated from peripheral blood samples from both donors and recipients using QIAamp DNA Blood-Mini Kit (Qiagen, Venlo, Netherlands), following the manufacturer’s recommendation.

### One-field HLA typing (=serological and low-resolution genetic HLA typing)

From 2015, serological typing was performed in-house using peripheral blood lymphocytes isolated by gradient centrifugation and typed for HLA-A, -B, and -C using commercial trays (inno-train GmbH, Kronberg, Germany) following the manufacturer’s protocol. HLA typing in one-field resolution was performed using PCR sequence specific priming (CareDx, San Francisco, CA, USA) following the manufacturer’s recommendations.

Prior to their transplantation, all patients on the kidney waiting list had been HLA-typed for HLA loci -A, -B, and -DR in line with the requirements of our registry and additional typing for -C and -DQ according to German Recommendations and in-hospital standard. At that time, results were recorded at split antigen level. Living donor HLA-typing data were registered in as well. For our assessment, we selected HLA data for donor/recipient pairs from the registry for HLA-A, -B, -C, DR, and -DQ, obtainable as one-field resolution at antigen level (i.e., serological or low-resolution molecular typing).

### Two-field HLA-typing (= NGS HLA typing)

Targeted sequencing of HLA genes was performed based on an NGS protocol using Illumina short read technology (SBS, sequencing by synthesis, San Diego, CA, USA), following the manufacturer’s recommendations. Initially a long-range PCR for the loci HLA-A, -B, -C, -DRB1, -DRB345, -DQA1, -DQB1, - DPA1, and -DPB1 was performed. Complete coding exons were covered for HLA-class I loci as well as for HLA-DQA1. For HLA-class II genes, exons 2-4 were amplified. Subsequently, the PCR products were purified using paramagnetic beads (MagSi-NGSprep Plus, Steinbrenner, Wiesenbach, Germany). Then a sequencing library was prepared using the QIAseq FX kit (Qiagen, Venlo, The Netherlands), which performs enzymatic fragmentation, end-repair, and adapter/index ligation. Sample-wise molecular barcoding was performed. We conducted sequencing on an Illumina Miseq machine using V2 chemistry (300 cycles). Data analysis was performed with the NGS-engine software using HLA-database V3.38 (GenDX, Utrecht, The Netherlands). Results were assigned using G-group nomenclature; non-expressed alleles were reported if present or excluded from the list of ambiguities. Here we use the term two-field for the definition of the respective typing, to compare the results with those of the Luminex data, where the information is given in the two-field nomenclature. This protocol was validated for low- and high-resolution clinical HLA typing and CE-marked as *in-vitro* diagnostic kit. In case a confirmatory typing was needed we used the AllType™ NGS assay provided by One Lambda, West Hills, CA, USA following the manufacturer’s recommendation.

### HLA antibody testing and epitope MM calculation

HLA antibody determination was performed on a Luminex^®^ platform using the LAB Screen™ assay provided by One Lambda, West Hills, CA, USA following the manufacturer’s recommendation. Screening for *de novo* DSA was routinely performed as part of the KT follow up,. i.e. at least every 3-6 months or if any sign of worsening KT function occurred (e.g. increase in serum creatinine or proteinuria). The analysis was performed using the Fusion software v 4.4. A mean fluorescence intensity (MFI) of ≥1500 was considered a positive finding. Additionally, it was analyzed to which extent HLA antibodies can be classified as “donor specific” based on the results of the two-field HLA typing (which means that without the results of the two-field typing, they would have been classified as non-donor-specific HLA antibodies). The detection of a donor-specific HLA antibody at a singular timepoint after KT was considered a “positive finding” and only such HLA antibodies that occurred *de novo* were considered in the evaluation. Epitope MM calculation for the case included in our manuscript was performed with HLA-Matchmaker algorithm using the epitope library 2020 (https://www.epregistry.com.br).

### Statistical analysis

To compare the results of the two different HLA typing methods (one-field vs. two-field) we generated separate MM counts for each donor/recipient pair and each typing technique. ‘**0**’ indicating, that donor and recipient have the same two alleles at an HLA locus. ‘**1**’ indicating, that for one HLA locus, the donor has one different HLA allele compared to the recipient. ‘**2**’ indicating, that the donor has two different HLA alleles compared to the recipient. That implies, that a minimum MM count of ‘0’ was possible, if donor and recipient shared the same alleles in all five HLA loci and a maximum of ‘10’ if both alleles of the HLA loci were distinct.

For data analysis and visualization, we used R (version 4.0.1; http://r-project.org), RStudio Desktop (version 1.3.959; http://rstudio.com), and Prism (version 9.4.1, GraphPad). We expressed results with 95% confidence interval. For normal distribution we performed Kolmogorov-Smirnov test. Statistical significance on MM counts was tested by a two-sided paired Student’s *t* test. The significance level was defined p ≤ 0.05 for two-tailed tests.

## Results

### Patients

A total of 143 living KT have been performed at the University of Leipzig Medical Center between 1998 und 2018. One patient (ind036) received subsequent kidney transplants from two living donors (ind053 ➔ pair-ID P114; ind177 ➔ pair-ID P5), explaining the different number of transplantations (n=143) and cohort size (n=142). More men than women received a living donor KT (male 59.9%, female 40.1%). The proportion of related donors was elevated (n=84; 58.7%). The recipients’ median age at chronic kidney failure and KT was 36.5 years and 38.4 years, respectively. The time between chronc kidney failure and living donor KT averages 35.1 months. 23 patients (16.2%) received a preemptive living donor KT, implying that no hemodialysis/peritoneal dialysis was performed prior to transplantation. 15 recipients (9.8%) received an AB0 incompatible KT. **Table 1** summarizes the characteristics of our cohort.

**Table 1:** Clinical and transplant characteristics of recipients and donors.

Clinical characteristics	Recipients (n=142)	Donors (n=143)
Age (y), mean ± SD	38.4 ± 16.6	49.6 ± 11.6
Sex (male), n (%)	85 (59.9)	55 (38.5)
European ancestry, n (%)	142 (100)	143 (100)
Age at chronic KF (y), mean ± SD	36.5 ± 15.4	
Time on dialysis (m), mean ± SD; range (m)	35.1 ± 38.1 1 – 208	
Preemptive KT (yes), n (%)	23 (16.2)	
Transplant characteristics		
Related donor (yes), n (%)	84 (58.7)	
1^st^ degree	61 (42.7)	
2^nd^ degree	19 (13.3)	
≥3^rd^ degree	4 (2.8)	
Donor/recipient sex, n (%)		
male ➔ female	29 (20.3)	
male ➔ male	26 (18.2)	
female ➔ female	28 (19.6)	
female ➔ male	60 (42.9)	
ABO incompatible KT, n (%)	15 (9.8)	
Recipients with *de novo* DSA, n (%)	50 (43.1)*	
class I	5 (4.3)	
class II	21 (18.1)	
class I & II	24 (20.7)	

Continuous variables are displayed as mean ± SD. DSA, donor specific antibodies; KF, kidney failure; KT, kidney transplantation; m, months; n; number, SD, standard deviation; y, years.

Categorial variables are displayed as n and (%). One patient (ind036) received subsequent kidney transplants from two living donors (ind053 ➔ pair-ID P114; ind177 ➔ pair-ID P5), explaining the different number of donors and recipients. *Of 127 donor/recipient pairs with 2-field HLA typing, 116 recipients had follow-up serum samples available for *de novo* DSA screening. Of these, 50 recipients developed *de novo* DSA after KT.

The most frequent causes of chronic renal failure were IgA nephropathy (24.7%) and congenital anomalies of the kidney an urinary tract (CAKUT, 17.6%), followed by patients in whom the etiology remained unknown (15.5%). **Supplementary Table ST1** further deciphers the underlying primary kidney diseases.

During post-transplant follow-up, a total of 58 recipients received at least one kidney transplant biopsy which was evaluated by experienced nephro-pathologists according to Banff classification (10). In 17 of these, histological assessment revealed evidence of acute and/or chronic ABMR.

Kidney transplant failure was observed in 12.6% (n=13) of 103 recipients during follow-up, including two patients who died during infectious/septic multiorgan failure, but who had prior stable renal graft function in each case. Among these patients with graft failure, 11 had at least one kidney transplant biopsy: acute ABMR n=2 (18.2%), acute cellular rejection n=2 (18.2%), both chronic ABMR and acute cellular rejection n=1 (9.1%), chronic ABMR n=3 (27.3%), no histologic signs of ABMR or cellular rejection n=3 (27.3%).

### HLA MM for one-field vs. two-field HLA typing (HLA-A, -B, -C, -DR, -DQ)

**Figure 2:**
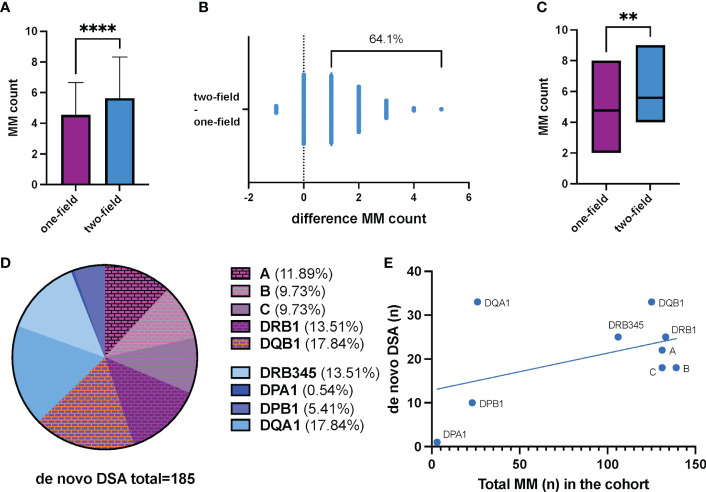
**(A)** Two-field HLA typing shows significantly more mismatches in our living kidney transplant cohort (n=103; median MM count low resolution HLA typing 4.54 ± 2.11; median MM count NGS HLA typing 5.63 ± 2.69; p<0.0001). Data represents the mean of the respective mismatch counts. The error bars indicate the standard error of the mean. **(B)** Illustration of the cumulative change in MM counts after NGS HLA typing compared to pre-transplantation one field HLA typing. Each dot represents a donor/recipient pair. Overall, 64.1% (n=66) showed an increased MM count (+1 to +5 additional MM). 33 pairs had a corresponding MM count. In 4 pairs, two-field HLA typing provided a lower MM count. **(C)** HLA MM count derived from one-field and two-field typing in patients with biopsy proven humoral renal transplant rejection. 58 KT recipients had at least one kidney graft biopsy during their post-KT period. Histological signs of acute and/or chronic humoral rejection were reported in 17 patients. Mismatch count by two-field HLA typing was significantly higher in these 17 patients compared to low resolution typing (median MM count NGS HLA typing 4.77 ± 1.17; median MM count NGS HLA typing 5.59 ± 1.73; p=0.0061). **(D)** Distribution of *de novo* DSA directed against the corresponding HLA loci. A total of 185 *de novo* DSA were found in our cohort. Among them, 69 (37.3%) were directed against HLA alleles and/or antigens not routinely considered in the allocation process by allocation organizations, so far. **(E)** Frequency of *de novo* DSA in relation to the number of HLA antigen/allele MM. A disproportionately high frequency of *de novo* DSA directed against DQA1 and DQB1 is observed. DSA, donor specific antibodies; MM, mismatch(es). ****p<0.0001; **p=0.0061.

103 donor/recipient pairs of our cohort had accessible one-field HLA typing data recorded in the registry for HLA-A, -B, -C, -DR, and -DQ. For all of them, two-field HLA typing delivered evaluable results. The mean calculated MM count for one-field and two-field HLA typing was 4.54 and 5.63, respectively (*t*-test p<0.0001). (**Figure 2A**) In 66 donor/recipient pairs (64.1%) two-field HLA typing revealed an increased MM count compared to recorded one-field HLA typing data. In 33 donor/recipient pairs (32.0%) the MM counts corresponded. In 4 donor/recipient pairs (3.9%) two-field HLA typing delivered a decreased HLA MM count. (**Figure 2B**) Two-field HLA typing unveiled a maximum of 5 additional HLA MM in one donor/recipient pair (pair-ID P55). In 9 recipients we identified a previously unknown maximal HLA MM count of 10 (**Supplementary Figure SF2**).

For the patients with ABMR (n=17), mismatch count by two-field HLA typing was significantly higher compared to one-field typing (median MM count NGS HLA typing 4.77 ± 1.17; median MM count NGS HLA typing 5.59 ± 1.73; p=0.0061). (**Figure 2C**) When analyzing each HLA locus separately, we show that in recipients with any graft rejection, i.e., both cellular and humoral graft rejection, unrecognized HLA mismatches were more likely to be located in HLA locus DQB1 and DRB1 (**Supplementary Figure SF3**).

### MM for HLA-DRB345, -DQA1, -DPA1, -DPB1 and de novo DSA

A rationale for allocation is to prevent major differences in HLA antigens or antigen groups between donor organ and recipient, since a higher number of MM increases the risk for the development of *de novo* DSA. Therefore, we aimed to assess to what extent the inclusion of additional HLA loci might be useful in allocation process. For this purpose, we analyzed the number of MM that we could only detect at the allele level in addition to the MM at antigen level. For each of the additional NGS typed HLA loci, MM at allele level were present in our cohort (**Table 2**).

**Table 2:** The number of both cumulative allele group and allele mismatches (n) in our cohort for each HLA locus as well as identified *de novo* donor-specific antibodies of allele and allele groups.

HLA Locus	Allele group mismatches (n)	Allele mismatches (n)	DSA of allele groups and alleles (n)	DSA identified upon HLA two-field typing (n (%))
A	128	3	22	0
B	136	3	18	0
C	114	17	18	1 (5.6%)
DQA1	–	26	33	3 (9.1%)
DQB1	98	27	33	3 (9.1%)
DRB1	131	2	25	0
DRB345	104	2	25	0
DPA1	–	3	1	0
DPB1	–	23	10	1 (10%)

Column 5 shows the number of *de novo* DSA (n (%)), that could only be identified with the information of two-field HLA typing.

In our cohort, 116 recipients had follow-up samples available for *de novo* DSA screening. *De novo* DSA developement was detected in 50 recipients (43.1%). Of these, 21 (18.1%) had HLA class II DSA, 5 (4.3%) had class I DSA, and 24 (20.7%) patients had both class I and II DSA. (**Table 1**) Considering the influence of HLA MM on *de novo* DSA formation, it was shown that DSAs were directed against all of these HLA loci of which testing in advance of transplantation is not necessarily required by our registry or national transplant organizations (HLA locus ➔ number of identified *de novo* DSA): DQA1 ➔ 33, DRB345 ➔ 25, DPA1 ➔ 1, DPB1 ➔ 10. (**Table 2**) Given the total number of n=185 *de novo* DSA for all HLA-loci, 37.3% (n=69) are directed against these additional tested HLA loci. (**Figure 2D**) No prior DSA were discarded based on 2-field typing.

## Discussion

Reducing graft immunogenicity through HLA allele matching is one of the cornerstones of organ transplantation. Histocompatibility assessment requires knowledge of the recipient’s anti-HLA antibody profile and the recipient’s and donor’s HLA phenotypes to reduce the risk of graft rejection due to memory and/or primary alloimmune reaction to mismatched donor HLA antigens (11, 12).

In this single-center study, we evaluated the impact of NGS-based two-field HLA typing in comparison to one-field HLA typing techniques in a cohort of living donor KT pairs. Thereby, we showed that two-field HLA typing is feasible and yields reliable results in all donor/recipient pairs. Here, two-field HLA typing significantly improves the detection of HLA MM compared to the data of one-field HLA typing for HLA loci -A, -B, -C, -DRB1, and -DQB1. Recently published work on cohorts with predominantly unrelated donor/recipient pairs replicates the finding of discrepant MM counts when comparing both one- and two-field typing for even three HLA loci (-A, -B, and -DRB1) (13). Considering that HLA antibodies may be reclassified as donor specific by uncovering typing discrepancies, this further emphasizes the application of two-field HLA typing techniques for optimized transplant management (13, 14).

Apart from the increased typing resolution of the NGS method itself, other factors could account for the difference in MM counts: First, inaccuracies in the context of a one-field HLA typing, especially serological typing, could be more prone to errors in implementation and interpretation. Interestingly, among those individuals with discrepancy in HLA MM counts, we observed 5 donor/recipient pairs. (**Supplementary Table ST2**) One explanation might be that donor/recipient pairs were HLA typed during a similar time period performed with the same lot of reagents, possibly leading to the confounding results. Second, as data transfer of HLA typing results is yet not fully automated, clerical errors on both sides (local and central) might have contributed to false entries as well. Overall, manual entry of such sensitive data seems outdated and no longer appropriate as manual data processing is generally associated with a high susceptibility to error (15). Fully digital interfaces could simplify the entry of HLA data in the future and reduce data errors. Taking into account that some patients on the waiting list for (deceased) KT have had their first HLA typing years ago, re-typing with two-field techniques should be considered to avoid discrepant typing data.

Our study demonstrates the presence of allele MM for additional typed HLA loci in a relevant number of donor/recipient pairs (DRB345, DPA1, DPB1 and DQA1). The number of MM is directly associated with the probability of *de novo* DSA development, which in turn is associated with increased risk of graft loss (4, 5, 16, 17). Likewise, we can demonstrate the presence of *de novo* DSA for all investigated HLA loci and a relevant proportion (37.3%) of them is directed against the HLA loci -DRB345, -DPA1, -DPB1, and -DQA1 (**Figure 2D**).

DQA1 accounts for the largest proportion (17.81%) of *de novo* DSAs directed against those HLA loci additionally typed. Recently, different studies outlined that DQ MM convey a significant impact on renal graft function and survival (18, 19). DQ antigens seem to have a previously underestimated role in the context of alloimmunity as the development of *de novo* anti-DQ DSA is associated with ABMR, transplant glomerulopathy, and graft loss (19–23).

Typing of the DRB345, DPA1, DPB1, and DQA1 is so far not necessary for organ allocation according to the National or Allocation Organization requirements and any translation of the results obtained in a living KT cohort to post-mortem organ donation is not straightforward. However, acknowledging that nearly 60% of our donor/recipient pairs were first-degree relatives, further studies should attempt to assess these findings in an unrelated postmortem KT donor cohort. The results of 2-field HLA typing did not lead to discarding of putative DSA of recipients. This contrasts previous studies with up to 20% discarded DSA (13). It is speculative whether this may be attributable to a higher proportion of living kidney transplants in our cohort and thus a higher likelihood of related recipient/donor pairs.

With respect to HLA-DRB1 and DRB345 the number of allele MM is lower than those for HLA-DQB1 and their respective HLA-DQA1. (**Table 2** annd **Figure 2E**) The production of DSA for HLA-DRB1, DRB345 seems to be directed towards the allele group rather than the allele itself, as shown for HLA-A and HLA-B (24), contrasting the results observed for HLA-C and HLA-DQB1/DQA1. (**Figure 2D**) The number of allele groups for HLA-C and HLA-DQB1 is low when typed on one-field or by serological methods, e.g., the allele group HLA-DQB1*03 comprises many alleles (DQB1*03:01 - DQB1*03:478) and for the allele group HLA-C*03 till now 589 alleles were reported (*IPD-IMGT/HLA database accessible at* http://hla.alleles.org). Whether the allele groups express similar or identical immunodominant epitopes is still a matter of further analysis. N.B., in the living transplant cohort analyzed here no restriction were done with respect to matching.

Additionally, two-field HLA typing is indispensable for the definition and calculation of HLA epitope MM, which cannot be identified by conventional serologic typing. Epitope MM rely on the differences of HLA amino acid sequences instead of entire HLA antigens (25). For allosensitization the consideration of epitope MM is reasonable, as anti-HLA antibodies recognize not a complete HLA antigen but different amino acid residues in antibody accessible regions (26–28). Already implemented by some transplant programs, epitope matching reduces DSA development and benefits graft survival in both deceased and living kidney donation (9, 11, 29–33). Epitope MM load is even associated with an increased risk of *de novo* DSA development after reduction of immunosuppression (26). Matching on epitope-level might therefore guide transplant physicians to identify those patients with the highest risk of *de novo* DSA development, who deserve tailored immunosuppressive regimens (9, 17, 26, 34, 35). Beyond, NGS methodology can further improve the calculation of non-synonymous single nucleotide polymorphisms outside the HLA coding regions, that were previously shown to be associated with allosensitization and allograft survival, as well (36).

Additionally, epitope analysis could mitigate post-secondary transplant complications in terms of risk reduction for antibody-mediated rejection and associated graft loss (see “Outlook/Case”). The presence of pre-transplant DSA critically determines longterm graft function and survival and the risk for ABMR (37). Therefore, epitope analysis based on 2-field HLA typing will further contribute to optimize organ allocation for (highly) sensitized patients on the KT waitlist. Better characterization of recipient antibody profiles helps identifying permissive mismatched donors (28, 32, 38–40). The focus on avoiding a small number of highly immunogenic epitope MM may in general be the optimal approach to enable access to organ allocation and concurrently minimizing the risk of allograft rejection (9, 17) However, the exclusive use of highly specific antibody detection methods, e.g., Luminex, without high-resolution HLA typing has the risk of rejecting whole antigens, even though allele-specificity of any HLA antibody is absent. In this context, before any implementation of a reliable virtual crossmatch a correct typing of the recipient is a prerequisite (a condictio sine qua non).

Our study has some limitations: First, we analyzed a living donor KT cohort, however, statements of this method in connection with living donation are difficult, since the donor selection is often very limited, meaning that often only one potential donor is available at all. Additionally, the influence of two-field HLA typing in deceased organ donation must be further elucidated, ideally in larger cohorts as the size of our cohort is in consideration of the recruitment period small. However, such studies seem only feasible, if the respective organ allocation organizations offer the possibility of two-field HLA data input. Manual input should be omitted. From a technical point of view, two-field HLA typing is suitable, as novel sequencing methods offer turnaround times of significantly less than 24 hours for 11 HLA loci (41, 42). From an economic point of view, high-resolution HLA typing by NGS has become a reasonable alternative, since low-resolution methods no longer offer any benefits and also costs for re-typing are eliminated (43).

Even if two-field HLA typing prior to deceased kidney donation cannot yet be regularly implemented in the context of kidney allocation, the objective should be to ensure subsequent high-resolution HLA typing after transplantation. Though it would not yet influence the allocation process itself, the results can still help to improve donor-specific antibody recognition and to provide a more personalized transplant management (44). In this regard, novel methods like DNA extraction from urinary cells are promising approaches for enabling HLA retyping even when donor DNA samples not available (45). In patients who are under evaluation of re-transplantation after graft failure, knowledge of the MM count is also helpful, as it has been shown that the number of panel reactive antibodies increases with each MM (46). In this context, it is particularly important to identify patients already sensitized by a previous transplantation using methods that are as sensitive as possible (37, 47, 48).

The setting of our study does not allow us for conclusions on the impact of high-resolution HLA typing on clinical parameters such as graft rejection or graft survival. Although MM count calculation revealed a significantly higher MM count for the two-field methodology in recipients with biopsy proven ABMR, this observation must be seen critically due to the single center character, the modest cohort size, and the fact that biopsies were not performed by protocol but by medical indication, e.g., increase in serum creatinine or proteinuria. In general, additional factors besides HLA matching influence graft survival in kidney transplantation, such as cold ischemia time or sufficient immunosuppressant levels. Therefore, the need for HLA matching in the context of living kidney transplantation must also always be seen as one among multiple contributing factors (7, 49).

Finally, our results support the implementation of two-field HLA typing results in solid organ transplant databases and Organ Procurement Organizations, to optimize organ allocation and to support personalized transplant medicine, as far as possible. Renewed genomic HLA typing of KT recipients with high resolution DNA sequencing methods is to be recommended as well as their electronic data transfer in the registry for avoidance of manual input errors.

### Outlook: Case report on the use of two-field typing in epitope analysis

**Figure 3:**
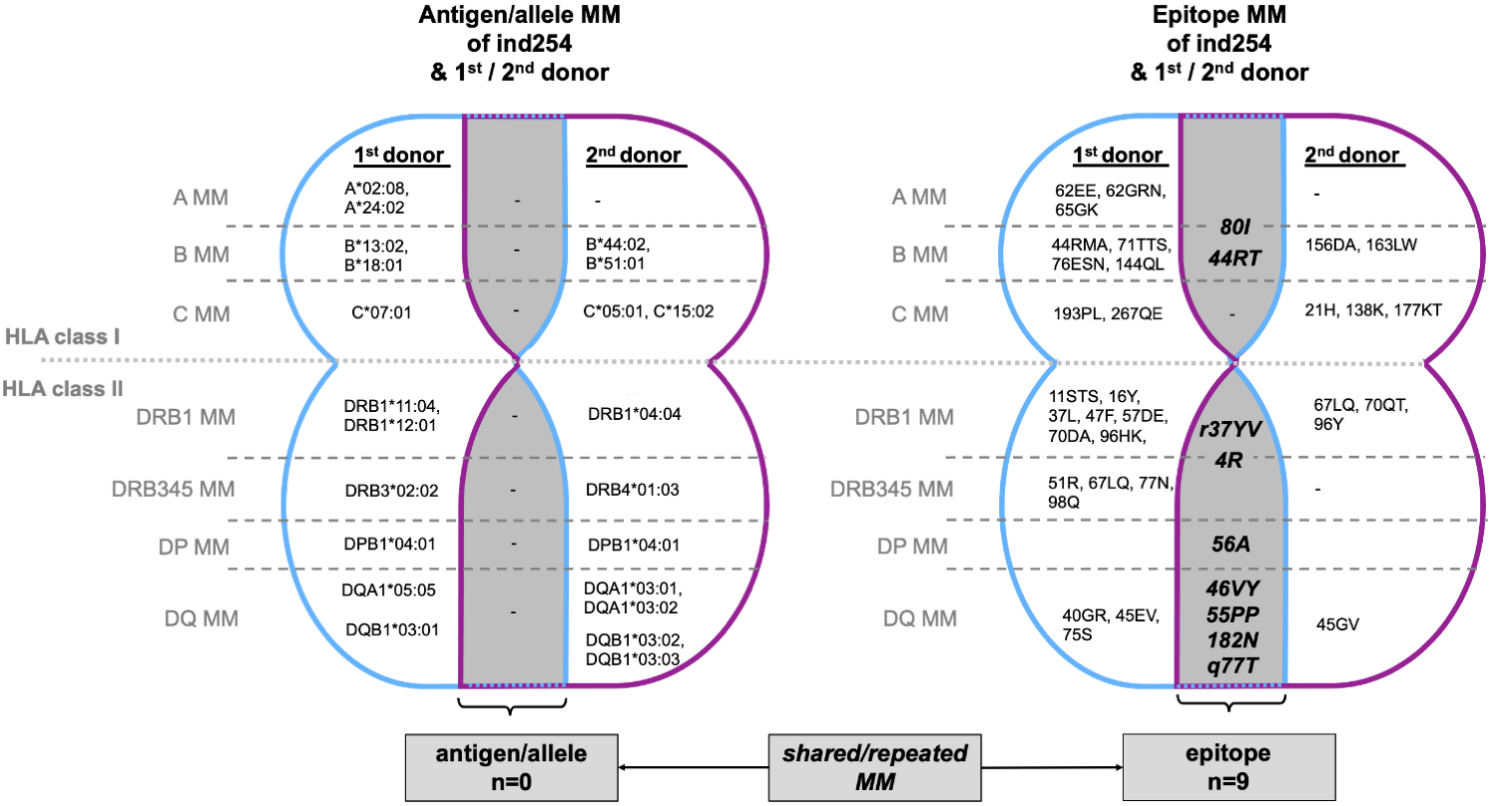
Case/Outlook: Illustraion of MM between KT recipient (ind254) and his 1^st^ (living) and 2^nd^ (postmortem) donor. The overlapping areas (gray) highlight those MM shared by the 1^st^ and 2^nd^ donors, i.e., MM that recur with the second transplantation. No repeated MM are observable at the antigen/allele level, but 9 MM at epitope level (HLA class I n=2, HLA class 2, n=7). Immunologically the immunization against the 2^nd^ donor, e.g., towards B*51:01 (epitopes 80I and 82LR) react against the A*24:02 of the primary donor. The comprehensive HLA typing of ind254 and both donors is deciphered in **Supplementary Table ST3**. HLA, human leukocyte antigen; MM, mismatch(es).

A 2-year-old patient (ind254) received a living kidney donation from his grandfather (P85). Due to graft failure, he returned to dialysis after 5 years. However, after another 6 years he was offered a postmortem kidney donation and was re-transplanted with a negative CDC crossmatch. Three weeks after the second KT the patient was diagnosed with biopsy proven acute ABMR. Epitope analysis based on (retrospective) two-field HLA typing revealed epitope incompatibilities, shared with both the first and second donor. (**Figure 3** and **Supplementary Table ST3**) The epitope mismatch analysis revealed 9 repeated MM for HLA class I and II in the second donor. Immunologically the immunization against the second donor, e.g., towards B*51:01 (epitopes 80I and 82LR) react against the A*24:02 of the primary donor. The B*51:01 allele of the second donor shares same epitopes with the B*18:01 (44RT) of the first donor. There is a variety of repeated epitope mismatches between the first and second donor in the B-Locus, which could even explain the reactivity of the B*78:01 bead in the Luminex SAB test. Detectable *de novo* DSAs are targeted to these epitopes, suggesting a gained sensitization during his first kidney transplant (**Supplementary Table ST4**), which remained undetected by the CDC crossmatch prior to his second transplantation.

Lessons to learn from the presented case is the necessity of two-field HLA typing of both recipients and donors and defining the epitope incompatibilities. The latter should be avoided when it comes to re-transplantation.

## Data availability statement

The datasets for this article are not publicly available due to concerns regarding participant/patient anonymity. Requests to access the datasets should be directed to the corresponding author.

## Ethics statement

The studies involving human participants were reviewed and approved by University Hospital Leipzig. Written informed consent to participate in this study was provided by the participants’ legal guardian/next of kin.

## Author contributions

SP, JM, KD, AW, AB, JdF, and FP gathered clinical data. CL, TL, RH, DF, and RL were responsible for genetic analysis. BP generated HLA analysis software. CL, ID, JH, and JM wrote the manuscript. CL, ID, JH, and JM conceived the study. All authors contributed to the article and approved the submitted version.

## References

[1] Tonelli M Wiebe N Knoll G Bello A Browne S Jadhav D. Systematic review: kidney transplantation compared with dialysis in clinically relevant outcomes. Am J Transplant (2011) 11:2093–109. doi: 10.1111/j.1600-6143.2011.03686.x 21883901

[2] Helanterä I Isola T Lehtonen TK Åberg F Lempinen M Isoniemi H. Association of clinical factors with the costs of kidney transplantation in the current era. Ann Transplant (2019) 24:393–400. doi: 10.12659/AOT.915352 31263093 PMC6625575

[3] Axelrod DA Schnitzler MA Xiao H Irish W Tuttle-Newhall E Chang S-H. An economic assessment of contemporary kidney transplant practice. Am J Transplant (2018) 18:1168–76. doi: 10.1111/ajt.14702 29451350

[4] Foroutan F Friesen EL Clark KE Motaghi S Zyla R Lee Y. Risk factors for 1-year graft loss after kidney transplantation: Systematic review and meta-analysis. Clin J Am Soc Nephrol (2019) 14:1642–50. doi: 10.2215/CJN.05560519 PMC6832056 31540931

[5] Lim WH Chadban SJ Clayton P Budgeon CA Murray K Campbell SB. Human leukocyte antigen mismatches associated with increased risk of rejection, graft failure, and death independent of initial immunosuppression in renal transplant recipients. Clin Transplant (2012) 26:E428–37. doi: 10.1111/j.1399-0012.2012.01654.x 22672477

[6] Summers DM Johnson RJ Allen J Fuggle SV Collett D Watson CJ. Analysis of factors that affect outcome after transplantation of kidneys donated after cardiac death in the UK: A cohort study. Lancet (2010) 376:1303–11. doi: 10.1016/S0140-6736(10)60827-6 20727576

[7] Opelz G. Impact of HLA compatibility on survival of kidney transplants from unrelated live donors. Transplantation (1997) 64:1473–5. doi: 10.1097/00007890-199711270-00017 9392314

[8] Tieken CM. Eurotransplant manual (2021). Available at: https://www.eurotransplant.org/wp-content/uploads/2020/01/H4-Kidney-2021.1-March-2021.pdf.

[9] Senev A Coemans M Lerut E van Sandt V Kerkhofs J Daniëls L. Eplet mismatch load and *De novo* occurrence of donor-specific anti-HLA antibodies, rejection, and graft failure after kidney transplantation: An observational cohort study. J Am Soc Nephrol (2020) 31:2193–204. doi: 10.1681/ASN.2020010019 PMC7461684 32764139

[10] Loupy A Haas M Roufosse C Naesens M Adam B Afrouzian M. The banff 2019 kidney meeting report (I): Updates on and clarification of criteria for T cell- and antibody-mediated rejection. Am J Transplant (2020) 20:2318–31. doi: 10.1111/ajt.15898 PMC7496245 32463180

[11] Lachmann N Niemann M Reinke P Budde K Schmidt D Halleck F. Donor-recipient matching based on predicted indirectly recognizable HLA epitopes independently predicts the incidence of *De novo* donor-specific HLA antibodies following renal transplantation. Am J Transplant (2017) 17:3076–86. doi: 10.1111/ajt.14393 28613392

[12] Senev A Lerut E van Sandt V Coemans M Callemeyn J Sprangers B. Specificity, strength, and evolution of pretransplant donor-specific HLA antibodies determine outcome after kidney transplantation. Am J Transplant (2019) 19:3100–13. doi: 10.1111/ajt.15414 31062492

[13] Meneghini M Perona A Crespo E Bemelman F Reinke P Viklicky O. On the clinical relevance of using complete high-resolution HLA typing for an accurate interpretation of posttransplant immune-mediated graft outcomes. Front Immunol (2022) 13:924825. doi: 10.3389/fimmu.2022.924825 36248818 PMC9559221

[14] Larkins NG D'Orsogna L Taverniti A Sharma A Chakera A Chan D. The accuracy of sequence-specific oligonucleotide and real-time polymerase chain reaction HLA typing in determining the presence of pre-transplant donor-specific anti-HLA antibodies and total eplet mismatches for deceased donor kidney transplantation. Front Immunol (2022) 13:844438. doi: 10.3389/fimmu.2022.844438 35799779 PMC9253866

[15] Barchard KA Pace LA. Preventing human error: The impact of data entry methods on data accuracy and statistical results. Comput Hum Behav (2011) 27:1834–9. doi: 10.1016/j.chb.2011.04.004

[16] Williams RC Opelz G McGarvey CJ Weil EJ Chakkera HA. The risk of transplant failure with HLA mismatch in first adult kidney allografts from deceased donors. Transplantation (2016) 100:1094–102. doi: 10.1097/TP.0000000000001115 PMC8086563 26901078

[17] Wiebe C Pochinco D Blydt-Hansen TD Ho J Birk PE Karpinski M. Class II HLA epitope matching-a strategy to minimize *de novo* donor-specific antibody development and improve outcomes. Am J Transplant (2013) 13:3114–22. doi: 10.1111/ajt.12478 24164958

[18] Lim WH Chapman JR Coates PT Lewis JR Russ GR Watson N. HLA-DQ mismatches and rejection in kidney transplant recipients. Clin J Am Soc Nephrol (2016) 11:875–83. doi: 10.2215/CJN.11641115 PMC4858494 27034399

[19] Sarabu N Hricik DE. HLA-DQ mismatching: Mounting evidence for a role in kidney transplant rejection. Clin J Am Soc Nephrol (2016) 11:759–60. doi: 10.2215/CJN.02970316 PMC4858473 27034401

[20] Willicombe M Brookes P Sergeant R Santos-Nunez E Steggar C Galliford J. *De novo* DQ donor-specific antibodies are associated with a significant risk of antibody-mediated rejection and transplant glomerulopathy. Transplantation (2012) 94:172–7. doi: 10.1097/TP.0b013e3182543950 22735711

[21] Ginevri F Nocera A Comoli P Innocente A Cioni M Parodi A. Posttransplant *de novo* donor-specific hla antibodies identify pediatric kidney recipients at risk for late antibody-mediated rejection. Am J Transplant (2012) 12:3355–62. doi: 10.1111/j.1600-6143.2012.04251.x 22959074

[22] Tagliamacco A Cioni M Comoli P Ramondetta M Brambilla C Trivelli A. DQ molecules are the principal stimulators of *de novo* donor-specific antibodies in nonsensitized pediatric recipients receiving a first kidney transplant. Transpl Int (2014) 27:667–73. doi: 10.1111/tri.12316 24629017

[23] Devos JM Gaber AO Teeter LD Graviss EA Patel SJ Land GA. Intermediate-term graft loss after renal transplantation is associated with both donor-specific antibody and acute rejection. Transplantation (2014) 97:534–40. doi: 10.1097/01.TP.0000438196.30790.66 24595116

[24] Vittoraki AG Fylaktou A Tarassi K Tsinaris Z Siorenta A Petasis GC. Hidden patterns of anti-HLA class I alloreactivity revealed through machine learning. Front Immunol (2021) 12:670956. doi: 10.3389/fimmu.2021.670956 34386000 PMC8353326

[25] Duquesnoy RJ. HLA epitope based matching for transplantation. Transpl Immunol (2014) 31:1–6. doi: 10.1016/j.trim.2014.04.004 24769079

[26] Snanoudj R Kamar N Cassuto E Caillard S Metzger M Merville P. Epitope load identifies kidney transplant recipients at risk of allosensitization following minimization of immunosuppression. Kidney Int (2019) 95:1471–85. doi: 10.1016/j.kint.2018.12.029 30955869

[27] El-Awar N Lee J-H Tarsitani C Terasaki PI. HLA class I epitopes: recognition of binding sites by mAbs or eluted alloantibody confirmed with single recombinant antigens. Hum Immunol (2007) 68:170–80. doi: 10.1016/j.humimm.2006.11.006 17349872

[28] Huang Y Dinh A Heron S Gasiewski A Kneib C Mehler H. Assessing the utilization of high-resolution 2-field HLA typing in solid organ transplantation. Am J Transplant (2019) 19:1955–63. doi: 10.1111/ajt.15258 30623581

[29] Philogene MC Amin A Zhou S Charnaya O Vega R Desai N. Eplet mismatch analysis and allograft outcome across racially diverse groups in a pediatric transplant cohort: a single-center analysis. Pediatr Nephrol (2020) 35:83–94. doi: 10.1007/s00467-019-04344-1 31599339 PMC6901410

[30] Tafulo S Malheiro J Santos S Dias L Almeida M La Martins S. Degree of HLA class II eplet mismatch load improves prediction of antibody-mediated rejection in living donor kidney transplantation. Hum Immunol (2019) 80:966–75. doi: 10.1016/j.humimm.2019.09.010 31604581

[31] Goodman RS Taylor CJ O'Rourke CM Lynch A Bradley JA Key T. Utility of HLAMatchmaker and single-antigen HLA-antibody detection beads for identification of acceptable mismatches in highly sensitized patients awaiting kidney transplantation. Transplantation (2006) 81:1331–6. doi: 10.1097/01.tp.0000205202.56915.f5 16699463

[32] Duquesnoy RJ Witvliet M Doxiadis II Fijter H Claas FH. HLAMatchmaker-based strategy to identify acceptable HLA class I mismatches for highly sensitized kidney transplant candidates. Transpl Int (2004) 17:22–30. doi: 10.1007/s00147-003-0641-z 12955350

[33] Sapir-Pichhadze R Zhang X Ferradji A Madbouly A Tinckam KJ Gebel HM. Epitopes as characterized by antibody-verified eplet mismatches determine risk of kidney transplant loss. Kidney Int (2020) 97:778–85. doi: 10.1016/j.kint.2019.10.028 32059998

[34] Daniëls L Claas FH Kramer CS Senev A Vanden Driessche M Emonds M-P. The role of HLA-DP mismatches and donor specific HLA-DP antibodies in kidney transplantation: a case series. Transpl Immunol (2021) 65:101287. doi: 10.1016/j.trim.2020.101287 32194154

[35] Kishikawa H Kinoshita T Hashimoto M Fukae S Taniguchi A Yamanaka K. Class II HLA eplet mismatch is a risk factor for *De novo* donor-specific antibody development and antibody-mediated rejection in kidney transplantation recipients. Transplant Proc (2018) 50:2388–91. doi: 10.1016/j.transproceed.2018.02.183 30316363

[36] Reindl-Schwaighofer R Heinzel A Kainz A van Setten J Jelencsics K Hu K. Contribution of non-HLA incompatibility between donor and recipient to kidney allograft survival: genome-wide analysis in a prospective cohort. Lancet (2019) 393:910–7. doi: 10.1016/S0140-6736(18)32473-5 30773281

[37] Frischknecht L Deng Y Wehmeier C Rougemont O Villard J Ferrari-Lacraz S. The impact of pre-transplant donor specific antibodies on the outcome of kidney transplantation - data from the Swiss transplant cohort study. Front Immunol (2022) 13:1005790. doi: 10.3389/fimmu.2022.1005790 36211367 PMC9532952

[38] Heidt S Witvliet MD Haasnoot GW Claas FH. The 25th anniversary of the eurotransplant acceptable mismatch program for highly sensitized patients. Transpl Immunol (2015) 33:51–7. doi: 10.1016/j.trim.2015.08.006 26325207

[39] Heidt S Haasnoot GW van Rood JJ Witvliet MD Claas FH. Kidney allocation based on proven acceptable antigens results in superior graft survival in highly sensitized patients. Kidney Int (2018) 93:491–500. doi: 10.1016/j.kint.2017.07.018 28947279

[40] Duquesnoy RJ Kamoun M Baxter-Lowe LA Woodle ES Bray RA Claas FH. Should HLA mismatch acceptability for sensitized transplant candidates be determined at the high-resolution rather than the antigen level? Am J Transplant (2015) 15:923–30. doi: 10.1111/ajt.13167 25778447

[41] Santis D Truong L Martinez P D'Orsogna L. Rapid high-resolution HLA genotyping by MinION Oxford nanopore sequencing for deceased donor organ allocation. HLA (2020) 96:141–62. doi: 10.1111/tan.13901 32274854

[42] Smith AG Pereira S Jaramillo A Stoll ST Khan FM Berka N. Comparison of sequence-specific oligonucleotide probe vs next generation sequencing for HLA-a, b, c, DRB1, DRB3/B4/B5, DQA1, DQB1, DPA1, and DPB1 typing: Toward single-pass high-resolution HLA typing in support of solid organ and hematopoietic cell transplant programs. HLA (2019) 94:296–306. doi: 10.1111/tan.13619 31237117 PMC6772026

[43] Stockton JD Nieto T Wroe E Poles A Inston N Briggs D. Rapid, highly accurate and cost-effective open-source simultaneous complete HLA typing and phasing of class I and II alleles using nanopore sequencing. HLA (2020) 96:163–78. doi: 10.1111/tan.13926 32419382

[44] Baxter-Lowe LA. Growing evidence that 2-field high-resolution HLA typing is important for kidney transplantation. Am J Transplant (2020) 20:3277–8. doi: 10.1111/ajt.16092 32484291

[45] Li X Wei Y Li J Deng R Fu Q Nie W. Donor HLA genotyping of ex vivo expanded urine cells from kidney transplant recipients. HLA (2021) 98:431–47. doi: 10.1111/tan.14426 34505410

[46] Meier-Kriesche H-U Scornik JC Susskind B Rehman S Schold JD. A lifetime versus a graft life approach redefines the importance of HLA matching in kidney transplant patients. Transplantation (2009) 88:23–9. doi: 10.1097/TP.0b013e3181a9ec89 19584676

[47] Bosch A Llorente S Eguia J Mrowiec A Boix F López-Hernández R. HLA-c antibodies are associated with irreversible rejection in kidney transplantation: Shared molecular eplets characterization. Hum Immunol (2014) 75:338–41. doi: 10.1016/j.humimm.2014.01.010 24486575

[48] Muro M González-Soriano MJ Salgado G López R Boix F López M. Specific "intra-allele" and "intra-broad antigen" human leukocyte antigen alloantibodies in kidney graft transplantation. Hum Immunol (2010) 71:857–60. doi: 10.1016/j.humimm.2010.05.018 20510320

[49] Terasaki PI Cecka JM Gjertson DW Takemoto S. High survival rates of kidney transplants from spousal and living unrelated donors. N Engl J Med (1995) 333:333–6. doi: 10.1056/NEJM199508103330601 7609748

