# Supplementary material for: Extended genomic HLA typing identifies previously unrecognized mismatches in living kidney transplantation
Source: Front Immunol. 2023 Jan 27;14:1094862. doi: 10.3389/fimmu.2023.1094862 (PMC9911689; doi:10.3389/fimmu.2023.1094862)
Supplement: Supplementary file 1 [file DataSheet_1.docx]

**SUPPLEMENTARY FILES:**

| **Kidney disease** | **n (%)** |
| --- | --- |
| IgA nephropathy | 35 (24.7) |
| CAKUT/ VUR/ chronic pyelonephritis | 25 (17.6) |
| unknown | 22 (15.5) |
| cystic kidney disease (ADPKD) | 16 (11.3) |
| hypertensive nephropathy | 8 (5.6) |
| ANCA vasculitis | 6 (4.2) |
| Alport syndrome | 4 (2.8) |
| FSGS | 4 (2.8) |
| HUS / aHUS | 4 (2.8) |
| hereditary kidney disease, other* | 3 (2.1) |
| membranous nephropathy | 3 (2.1) |
| lupus nephritis | 2 (1.4.) |
| toxic nephropathy | 2 (1.4) |
| interstitial nephritis | 2 (1.4) |
| diabetic nephropathy | 1 (0.7) |
| immune complex glomerulonephritis | 1 (0.7) |
| minimal change glomerulonephritis | 1 (0.7) |
| anti-GBm syndrome | 1 (0.7) |
| nephrectomy | 1 (0.7) |
| fibrillary glomerulonephritis | 1 (0.7) |
| total | 142 |

**Supplementary Table ST1**

**Supplementary Table ST1:** Renal diseases that resulted in end stage kidney disease. Of note, a kidney disease was only specified when it was proven by renal biopsy or reliable results from genetic diagnostics. The diagnosis ‘ADPKD’ was substantiated either on genetic diagnostics or results from renal imaging. *’hereditary kidney disease, other’ subsumes patients with pathogenic variants in *WT1* (OMIM *607102), *SMARCAL1* (OMIM*606622) und *AGXT* (OMIM *259900). Abbreviations: ADPKD; autosomal dominant polycystic kidney disease; aHUS, atypical hemolytic uremic syndrome; ANCA, anti-neutrophil cytoplasmatic antibodies; CAKUT, congenital anomalies of the kidneys and urinary tract; FSGS focal-segmental glomerulosclerosis; GBm, glomerular basement membrane; HUS, hemolytic uremic syndrome; OMIM, online mendelian inheritance in man. VUR, vesicoureteral reflux.

**Supplementary Table ST2**

| ID (Pair-ID) | Donor / Recipient | HLA-Locus Discrepancy | One-field  HLA-Typing | Two-field  HLA-Typing |
| --- | --- | --- | --- | --- |
| **ind001** (P1) | D | DRB1 | DR3  DR13 | DRB1*03:01:01  DRB1*12:01:01 |
| **ind002** (P1) | R | C  DQB1 | Cw6  Cw7  DQ1  DQ3 | C*06:02:01  C*17:01:01  DQB1*03:01:01  DQB1*03:02:01 |
| **ind005** (P100) | D | B | B18  B57 | B*18:01:01  B*58:02:01 |
| **ind006** (P100) | R | C | Cw2  Cw7 | C*02:02:02  C*15:05:01 |
| **ind081** (P135) | R | A | A*25  A*69 | A*25:01:01  - |
| **ind082** (P135) | D | A | A*25  A*69 | A*25:01:01  - |
| **ind111** (P2) | R | B | B8  B38 | B*07:02:01  B*38:01:01 |
| **ind112** (P2) | D | B | B7  B8 | B*07:02:01  B*38:01:01 |
| **ind121** (P24) | D | C | Cw4 | C*04:01:01  C*05:01:01 |
| **ind134** (P34) | D | A  C | A2  A30  Cw7 | A*02:01:01  A*02:05:01  C*06:02:01  C*15:05:01 |
| **ind138** (P31) | R | C | Cw6 | C*03:04:01  C*06:02:01 |
| **ind152** (P38) | R | DRB1  DRB345 | DR3  DR14  DR52 | DRB1*07:01:01  DRB1*10:01:01  DRB4*01:01:01 |
| **ind160** (P41) | D | C | Cw7 | C*02:02:02  C*07:02:01 |
| **ind167** (P45) | R | DQB1 | DQ3(8) | DQB1*03:01:01  DQB1*03:02:01 |
| **ind218** (P69) | R | C | Cw5 | C*05:01:01  C*06:02:01 |
| **ind231** (P74) | D | DRB1  DRB345  DQB1 | DR4  DR13  DR53  DQ3  DQ3 | DRB1*03:01:01  DRB1*08:01:01  DRB3*02:02:01  DQB1*02:01:01  DQB1*04:02:01 |
| **ind232** (P75) | R | A | A3  A23 | A*03:01:01  A*24:02:01 |
| **ind247** (P81) | D | A | A24 | A*24:02:01  A*33:03:01 |
| **ind261** (P88) | D | DQB1 | DQ6 | DQB1*03:01:01  DQB1*06:03:01 |
| **ind268 (**P91) | D | A | A23  A24 | A*24:02:01  - |
| **ind269** (P91) | R | C | Cw2  Cw7 | C*02:02:02  C*15:02:01 |

**Supplementary Table ST2:** Detailed information of previous HLA typing results that were recorded in the registry (one-field HLA typing) and results from two-field HLA typing which delivered discrepant results. Of note, 10/21 patients belong to a corresponding donor/recipient pair (P1, P2, P91, P100, P135). Abbreviations: D, donor; R, recipient.

**Supplementary Table ST3:**

|  | **Patient** | **1^st^ donor** | **2^nd^ donor** |
| --- | --- | --- | --- |
| **HLA class I** | A*01:01, A*02:01  B*44:03, B*47:01  C*06:02, C*16:01 | A*02:08, A*24:02  B*13:02, B*18:01  C*06:02, C*07:01 | A*02:01, A*02:01  B*44:02, B*51:01  C*05:01, C*15:02 |
| A MM |  | 62EE, 62GRN, 65GK, 80I | - |
| B MM |  | 44RMA, ***44RT***, 71TTS, 76ESN, 144QL | ***44RT***, 80I, 156DA, 163LW |
| C MM |  | 193PL, 267QE | 21H, 138K, 177KT |
|  | | | |
| **HLA class II** | DRB1*07:01, DRB1*09:01  DRB4*01:01, -  DQA1*02:01, DQA1*03:03  DQB1*02:01, -  DPA1*01:03, DPA1*02:01  DPB1*02:01, DPB1*17:01 | DRB1*11:04, DRB1*12:01  DRB3*02:02, -  DQA1*05:05, -  DQB1*03:01, -  DPA1*01:03, -  DPB1*02:01, DPB1*04:01 | DRB1*04:04, DRB1*09:01 DRB4*01:03, -  DQA1*03:01, DQA1*03:02  DQB1*03:02, DQB1*03:03  DPA1*01:03, -  DPB1*02:01, DPB1*04:01 |
| DRB1 MM |  | ***4R***, 11STS, 16Y, 37L, 47F, 57DE, 70DA, 96HK, ***r37YV*** | ***4R***, 67LQ, 70QT, 96Y, ***r37YV*** |
| DRB345 MM |  | ***4R*,** 51R, 67LQ, 77N, 98Q | - |
| DP MM |  | ***56A*** | ***56A*** |
| DQ MM |  | 40GR, 45EV, ***46VY****,* ***55PP***, 75S, ***182N****,* ***q77T*** | 45GV, ***46VY****,* ***55PP****,****182N****,* ***q77T*** |

**Supplementary Table ST3**: Epitope mismatches (MM) for HLA class I (upper table) and HLA class II (lower table) as an example for patient ind254 and his two kidney transplant donors. Repeated Epitope MM between first (P85) and second donor are indicated in **bold** and *italic*. There is 1 repeated epitope MM for HLA class I and 7 repeated epitope MM for HLA class II. Immunologically the immunization against the second donor, e.g., towards B*51:01 (epitopes 80I and 82LR) react against the A*24:02 of the primary donor. The B*51:01 allele of the second donor shares same epitopes with the B*18:01 (44RT, 69TNT, 131S) and B*13:02 (69TNT, 131S) of the first donor. There is a variety of repeated epitope MM between the first and second donor in the B-locus, which could even explain the reactivity of an B*78:01 bead in the Luminex SAB test.

**Supplementary Table ST4:**

|  | **HLA class I** | | **HLA class II** | |
| --- | --- | --- | --- | --- |
|  | **Antibodies** | **Epitopes** | **Antibodies** | **Epitopes** |
| **Before 2^nd^ KT** (crossmatch serum) | - | - | DR8, ***DR11, DR12,*** DR13, DR14, DR16, DR17, DR18, ***DR52, DQ7,*** DQ8, DQ9, DQA1*03:01, DQA1*03:02, DQA1*05:03, ***DQA1*05:05,*** DQA1*06:01 | ***96HK***, ***55PP***, ***11STS***, ***77N, 45EV***, ***74R***, ***16Y***, ***98Q***, ***57DE***, ***37L***, ***51R*** |
| **After 2^nd^ KT** | A23, ***A24,*** **B51,** B78 | ***65GK*** | **DR4**, DR8, ***DR11,*** ***DR12,*** DR13, DR14, DR15, DR16, DR17, DR18, DR51, ***DR52,*** DQ4, DQ5, DQ6, ***DQ7,* DQ8**, **DQ9**, DQA1*01:01, DQA1*01:02, DQA1*01:03, DQA1*03:01, DQA1*03:02, DQA1*05:01, DQA1*05:03, ***DQA1*05:05*,** DQA1*06:01, DP11 | 46VY, q77T, 45GV, 182N, 55R, ***96HK***, ***55PP***, ***11STS***, 47F, r37YV, 52PQ, 52SK, 74S, 142M, ***45EV***, 96Y, ***77N***, 87F, 87Y, 56L, ***74R***, ***98Q***, q57V, ***16Y***, 75S, 57S, ***37L***, ***57DE***, 116I, ***51R***, 125SQ |

**Supplementary Table 4**: HLA antibody reactivity of the patient ind254 before and after second kidney transplantation. **Bold** and *italic* indicate the DSA against the 1^st^ donor (P85) and the corresponding positive epitopes. Bold and underlined indicate de novo DSA against the 2^nd^ donor and the corresponding positive epitopes

**SUPPLEMENTARY FIGURES:**

**Supplementary Figure SF1**

Illustration of mismatch counts obtained from the results of one-field typing recorded in ENIS (low-resolution and serological) and two-field HLA re-typing for each donor/recipient pair (Y-axis indicates the number of mismatches. X-axis indicates the donor/recipient pairs. Green dots represent the cumulative mismatch count for one-field typing, blue rhombuses those for two-field typing). The corresponding mismatch count for the HLA locus is given in brackets (A, B, C, DRB1, DQB1). In 66 donor/recipient pairs (64.1 %) two-field HLA typing revealed an increased mismatch count compared to recorded low resolution HLA typing. In 33 donor/recipient pairs (32.0 %) the mismatch counts corresponded. In 4 donor/recipient pairs (3.9 %) two-field HLA typing delivered a decreased HLA mismatch count. Two-field HLA typing unveiled a maximum of 5 additional HLA mismatches in one donor/recipient pair (pair-ID P55).

**Supplementary Figure SF2:**

**A.** Based on the typing methods, this figure illustrates the distribution of the calculated mismatch counts achieved with one-field typing (green) and two-field typing (blue). **B.** In the assessment of the existing allele mismatches in our cohort, it becomes apparent that for loci that normally cannot be submitted to the registry and thus are not considered for the allocation of kidney grafts, a relevant number of mismatches is present. Typing for these loci in the context of allocation, however, seems reasonable, because at least in our cohort a corresponding number of de novo donor-specific antibodies (DSA) could be detected. Based on the two-field typing data, we could also show that 4.3% of the de novo DSA can only be identified as such with the help of high-resolution typing

**Supplementary Figure SF3:** When analyzing each HLA locus separately, we show that in recipients with any graft rejection (including both cellular and humoral graft rejection), unrecognized HLA mismatches were more likely to be located in HLA locus DQ (= DQB1) and DR (= DRB1)

**Supplementary Figure SF1:**

**Supplementary Figure SF2:**

**Supplementary Figure SF3:**
